# A programmed cell death-related model based on machine learning for predicting prognosis and immunotherapy responses in patients with lung adenocarcinoma

**DOI:** 10.3389/fimmu.2023.1183230

**Published:** 2023-08-21

**Authors:** Yi Zhang, Yuzhi Wang, Jianlin Chen, Yu Xia, Yi Huang

**Affiliations:** ^1^ Shengli Clinical Medical College of Fujian Medical University, Fujian Medical University, Fuzhou, Fujian, China; ^2^ Department of Clinical Laboratory, Fujian Provincial Hospital, Fuzhou, China; ^3^ Department of Laboratory Medicine, Deyang People’s Hospital, Deyang, Sichuan, China; ^4^ Integrated Chinese and Western Medicine College, Fujian University of Traditional Chinese Medicine, Fuzhou, Fujian, China; ^5^ Central Laboratory, Center for Experimental Research in Clinical Medicine, Fujian Provincial Hospital, Fuzhou, China; ^6^ Fujian Provincial Key Laboratory of Critical Care Medicine, Fujian Provincial Key Laboratory of Cardiovascular Disease, Fuzhou, China

**Keywords:** programmed cell death, lung adenocarcinoma, machine learning, prognosis, tumor microenvironment

## Abstract

**Background:**

lung adenocarcinoma (LUAD) remains one of the most common and lethal malignancies with poor prognosis. Programmed cell death (PCD) is an evolutionarily conserved cell suicide process that regulates tumorigenesis, progression, and metastasis of cancer cells. However, a comprehensive analysis of the role of PCD in LUAD is still unavailable.

**Methods:**

We analyzed multi-omic variations in PCD-related genes (PCDRGs) for LUAD. We used cross-validation of 10 machine learning algorithms (101 combinations) to synthetically develop and validate an optimal prognostic cell death score (CDS) model based on the PCDRGs expression profile. Patients were classified based on their median CDS values into the high and low-CDS groups. Next, we compared the differences in the genomics, biological functions, and tumor microenvironment of patients between both groups. In addition, we assessed the ability of CDS for predicting the response of patients from the immunotherapy cohort to immunotherapy. Finally, functional validation of key genes in CDS was performed.

**Results:**

We constructed CDS based on four PCDRGs, which could effectively and consistently stratify patients with LUAD (patients with high CDS had poor prognoses). The performance of our CDS was superior compared to 77 LUAD signatures that have been previously published. The results revealed significant genetic alterations like mutation count, TMB, and CNV were observed in patients with high CDS. Furthermore, we observed an association of CDS with immune cell infiltration, microsatellite instability, SNV neoantigens. The immune status of patients with low CDS was more active. In addition, CDS could be reliable to predict therapeutic response in multiple immunotherapy cohorts. *In vitro* experiments revealed that high DNA damage inducible transcript 4 (*DDIT4*) expression in LUAD cells mediated protumor effects.

**Conclusion:**

CDS was constructed based on PCDRGs using machine learning. This model could accurately predict patients’ prognoses and their responses to therapy. These results provide new promising tools for clinical management and aid in designing personalized treatment strategies for patients with LUAD.

## Introduction

Globally, lung cancer (LC) accounts for approximately 18% of all cancer-related mortalities and is also the leading cause of cancer-related mortalities in both sexes (1). Non-small cell LC (NSCLC) accounts for 90% of LC cases. NSCLC can be further categorized based on histology into two subtypes: lung adenocarcinoma (LUAD) and lung squamous cell carcinoma (LUSC). Of these, LUAD cases are more common (2). The factors underlying the poor prognosis of patients with mid to late-stage LUAD include the lack of symptoms and tumor specificity at an early stage, local infiltration, and distant metastases of cancer (3). Rapid advancements in biotechnology and precision medicine have helped develop targeted drugs and therapeutic approaches specific to patients with LUAD. Further biomarkers for LUAD, like *EGFR, E17K*, and *PTEN*, have been identified (4–6), which are currently used in combination with surgical resection, radio, and chemotherapies. However, only a small proportion of patients with LUAD have benefitted from these advancements and improvements in therapeutic efficacy. No significant improvement in the overall survival (OS) and progression-free survival of patients has been observed (7, 8). Therefore, an in-depth understanding of the underlying mechanisms of LUAD and identifying new biomarkers is crucial for predicting the prognoses and designing personalized therapeutic strategies for patients with LUAD.

Programmed cell death (PCD) is a crucial process for the growth and development of living organisms. Studies have shown that apoptosis, pyroptosis, ferroptosis, autophagy, necroptosis, cuproptosis, parthanatos, entotic and lysosome-dependent cell death, Alkaliptosis, NETosis, and oxeiptosis-related PCDs are classical cell death pathways (9). Apoptosis is a non-inflammatory response to PCD characterized by the activation of caspases, leading to the contraction of cells, coalescence, and the nucleus, as well as nucleosomal DNA fragmentation (10). Apoptosis is required for the maintenance of the cell death-cell survival balance. Furthermore, abnormal apoptosis escape is a characteristic of cancer cells (11). Pyroptosis is programmed necrosis of cells induced by inflammatory vesicles, wherein activated Gasdermin protein (a scorching substrate for inflammatory caspases-1/4/5/11) forms pores in the plasma membrane, thereby leading to cell death (12, 13). In 2012, ferroptosis was discovered as a novel iron-dependent PCD characterized by its ability to disrupt the redox homeostasis of cells and the absence of apoptosis (14). During ferroptosis, the cytoplasm appears round and detached, the mitochondrial membranes are condensed, the number of mitochondrial cristae is reduced or absent, and the outer mitochondrial membranes are ruptured (15). Autophagy is an apoptosis-independent cell death form. It is characterized by no chromatin condensation, the accumulation of autophagic vacuole, and autophagosome formation, which fuses with lysosomes to form autolysosomes in the cytoplasm (16, 17). Unlike apoptosis, necroptosis destabilizes cell membranes, and cause swelling and lysis of cells, thereby leading to the release of cellular components (18). The inactivation or deletion of caspases-8 and RIPK1 and RIPK3 activation, as well as autophosphorylation, induces necroptosis of cells (19). During necroptosis, the cell membranes rupture and release cellular contents, thereby activating immune responses (20). In March 2022, a study by Peter et al. introduced a new mode of cell death called cuproptosis (21). Unlike other forms of cell death, copper toxicity occurs primarily through the direct binding of cuproptosis to the fatty acylated components of the Krebs cycle. This leads to fatty acylated protein accumulation and iron-sulfur cluster protein loss, increase in proteotoxicity, which culminates in cell death (22). Parthantos is characterized by an increase in the activation of PARP-1 (23), PAR aggregates, and the translocation of apoptosis-inducing factors from the mitochondria to the nucleus (24). Unlike pyroptosis, parthanatos is independent of caspase and is triggered by an excessive reactive oxygen species (ROS) response (25). A study has shown that parthanatos induces mitochondrial membrane dissipation and the condensation of extensively fragmented DNA chromatin (26). Entotic cell death is the byproduct of endocytosis, forms typical intercellular structures, and is caused by the disassociation of cells from the basement membrane. It primarily occurs in epithelial cells and carcinomas (27). The entry of epithelial cells into other cells can eliminate endosomal cells by specific autophagy-related processes regulating the lysosomal degradation of cells (28). NETosis is a type of neutrophils, granulocytes, or macrophage-related necrosis. During NETosis, the granular contents of neutrophils are transferred to the nucleus, which causes the decondensation of chromatins, and induces the formation of a neutrophil extracellular trap (29). Lysosomal membrane permeabilization (LMP) is the primary cause of lysosome-dependent cell death, characterized by the loss of the lysosomal membrane integrity, thereby releasing the contents of lysosomes into the cytosol (30). LMP-mediated cell death is either dependent or independent of caspases (31). Alkalinization in cells induces a novel mode of PCD called alkaliptosis (32). The oxygen radicals trigger a novel form of regulated cell death called oxeiptosis, which is independent of caspases, and driven by the KEAP1-PGAM5-AIFM1 pathway activation (33). In organisms, PCD eliminates harmful or redundant cells and maintains tissue homeostasis. During PCD, damage-associated molecular patterns are released, which act as a powerful stimulus for activating local inflammatory or systemic immune responses. Therefore, selective activation of the PCD pathway could be a novel strategy for preventing and treating patients with LUAD. A study has shown that A549 cells treated with chemotherapeutic drugs such as cisplatin and paclitaxel trigger pyroptosis *via* the caspase 3/Gasdermin E pathway. The efficacy of these drugs to stimulate pyroptosis depends on the expression of Gasdermin E (34). CD8+ T cells secrete IFNs, which reduce SLC7A11 and SLC3A2 expression, thereby preventing the uptake of cystine by LUAD cells and promoting ferroptosis as well as lipid peroxidation. Together, this enhances the efficacy of immunotherapy. Hydroxychloroquine inhibits LUAD cell autophagy, thereby reversing chemoresistance in advanced-stage LUAD (35). Thus, escaping multiple types of PCD is a hallmark of LUAD. Therefore, a comprehensive understanding of the underlying mechanism of pan-PCD in LUAD could aid in mitigating tumorigenesis, cancer progression, and drug resistance in LUAD.

Previous studies on PCD have determined the involvement of a single mode of cell death in LUAD. However, several modes of PCDs mediate tumorigenesis, progression, and metastasis of cancer cells. Moreover, no studies have analyzed the involvement of PCD in LUAD systematically. In this study, we investigated the alterations of PCD-related genes (PCDRGs) in LUAD. We used a computational framework to construct and validate a novel cell death score (CDS) based on PCDRGs. CDS can accurately stratify patients with LUAD based on their prognostic status. Next, we investigated the differences in genetic mutations, tumor microenvironment (TME), and biological characteristics of patients between both CDS groups. Furthermore, we determined the efficiency of CDS in predicting the patients’ responses to immunotherapy and screened suitable drugs for patients with LUAD in different CDS groups. Finally, we determined the roles of DNA damage inducible transcript 4 (*DDIT4*) in LUAD.

## Materials and methods

### Cohort and preprocessing

**Figure 1:**
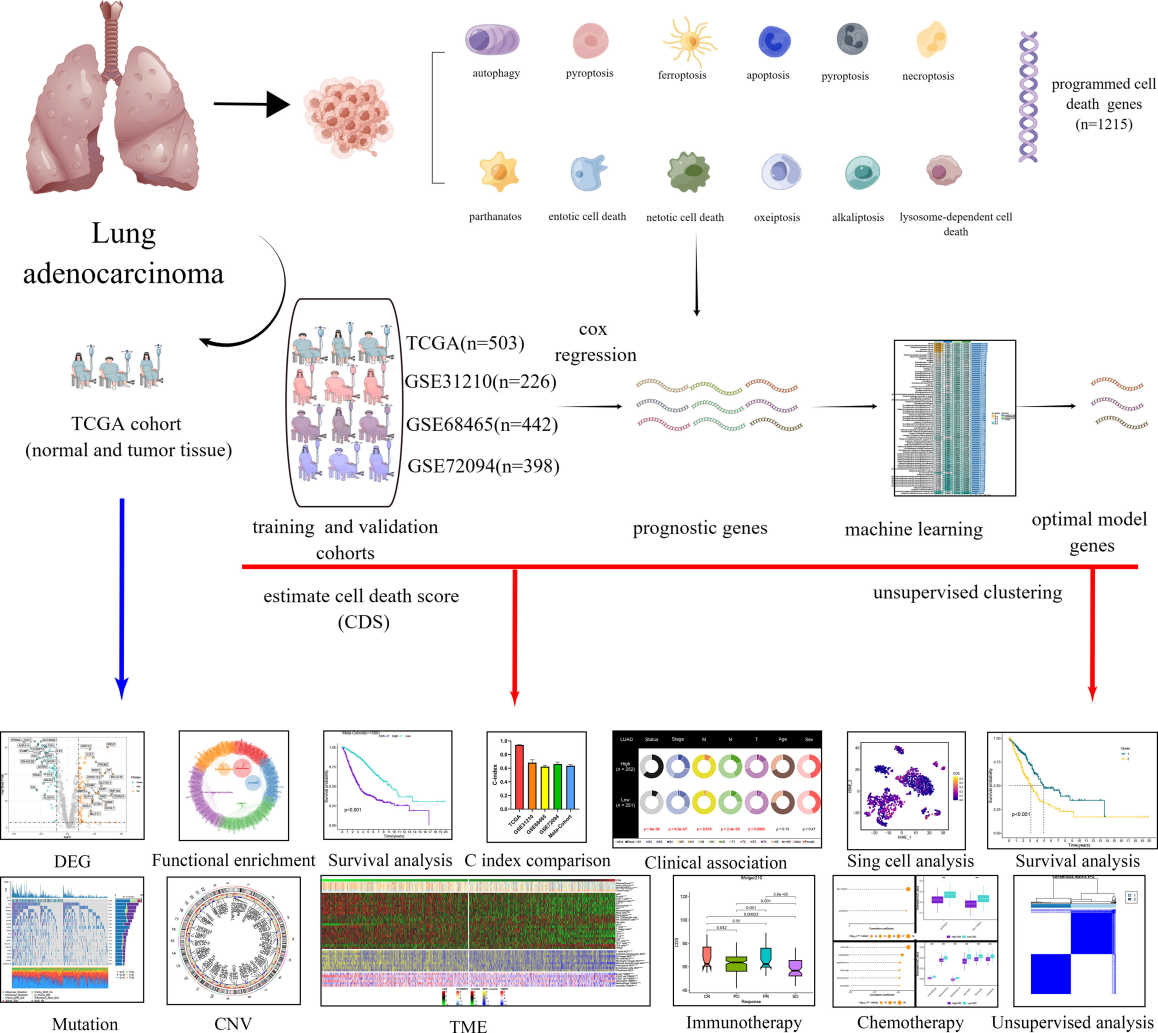
Diagram of analytic workflow in this study. The Diagram was drawn from the FIGUREdraw. (https://www.FIGUREdraw.com/static/index.html).

The transcriptional and clinical data of patients with LUAD were downloaded from the Cancer Genome Atlas (TCGA, https://portal.gdc.cancer.gov/) and Gene Expression Omnibus (GEO, http://www.ncbi.nlm.nih.gov/geo) databases. In addition, the data on gene mutation and copy number variation (CNV) were obtained from TCGA database. Next, we merged four cohorts, and the batch effect was eliminated using the “Combat” algorithm. The TCGA-LUAD cohort was used as the training cohort for constructing the CDS. The GSE31210, GSE68465, and GSE72094 cohorts from GEO were used as independent validation cohorts. We excluded patients whose OS information was unavailable. Finally, we included 1569 patients with LUAD for the subsequent analysis. **Supplementary Table 1** shows detailed information on the patients. The study flowchart is depicted in **Figure 1**.

### The expression and variations of PCDRGs in LUAD

The key regulatory genes of these 12 types of PCD were included as PCDRGs. These genes were collected and compiled from the Gene Set Enrichment Analysis (GSEA), Kyoto Encyclopedia of Genes and Genomes (KEGG), and previously published gene sets (9) (**Supplementary Table 2**). The differentially expressed PCDRGs (DEPCDRGs) in LUAD and paracancerous tissues of patients in the TCGA-LUAD cohort were screened using the “limma” R package. The threshold for screening DEPCDRGs was “*P* < 0.05” and “|log2 Fold change (FC)| > 1”. The functional enrichment analysis was performed to identify functions and pathways enriched by DEPCDRGs using the “clusterprofiler” R package. Next, the “maftools” package was employed to explore the somatic mutations in DEPCDRGs (36). The frequencies of “Gain” or “Loss” CNV in DEPCDRGs were screened and calculated. Finally, the chromosomal location of CNV in patients was visualized as the circus plot with the aid of the “circlize” R package (37). The transcription factors (TFs) within the DEPCDRGs were predicted using Transcriptional Regulatory Relationships Unraveled by Sentence-based Text mining (TRRUST, www.grnpedia.org/trrust/). Subsequently, TF-gene interaction pairs exhibiting P-values <0.05 were carefully chosen to construct the regulatory network through the utilization of Cytoscape.

### PCDRGs signature generated by machine learning-based integrated approach

The prognosis-related DEPCDRGs were screened using the univariate Cox regression analysis. The threshold set to avoid omission was “*P* < 0.05”. A PCDRGs signature was constructed with high accuracy and stability using 10 machine learning algorithms, including “Least Absolute Shrinkage and Selection Operator”, “Ridge”, “Elastic network”, “StepCox”, “Survival support vector machine (survival-SVM)”, “CoxBoost”, “Supervised principal components”, “partial least squares regression for COX”, “random survival forest (RSF)”, “generalized boosted regression modeling” to construct. These 10 machine learning algorithms were used to cross-validate 101 combinations of the “leave-one-out-cross validation (LOOCV)” framework for constructing the models based on the TCGA-LUAD cohort and were subsequently validated in the GEO cohorts. The models with < 3 genes were excluded. Additionally, we calculated the C-index of each model in all cohorts. CDS was the optimal model with the highest mean C-index. All patients were classified using the median CDS value set as a threshold into the high and low-CDS groups.

### Consensus clustering

Consensus clustering was performed using the “ConsensusClusterPlus” package based on the expression of PCDRGs in the CDS. The clustering was based on dividing centromeres with “Euclidean” distances. Finally, patients with LUAD were classified into two subtypes based on the best classification of “K=2-9”.

### Mutation and CNV characteristics

The mutation profiles and types of the top 20 genes with the highest mutation frequencies in patients in both CDS groups were mapped using the “maftools” package. Tumor mutational burden (TMB) is the total number of non-synonymous mutations in all exomes of patients and is calculated according to the number of non-synonymous mutations/million bases. Simultaneous analysis of significantly mutated genes and their interactions among mutations between two CDS groups. “GISTIC 2.0” was employed for screening significantly amplified and missing genomic regions (38). The overall changes in the genome were quantified by calculating fraction of genome alteration (FGA), fraction of genome gained (FGG), and fraction of genome lost (FGL). FGA is the percentage of fragmented bases of genomic variants. FGG/FGL indicated the loss or gain of genomic variants.

### TME annotations for CDS

Single Sample Gene Set Enrichment Analysis (ssGSEA), Tumor IMmune Estimation Resource (TIMER), and “MCPcounter” were used estimate the extent of immune cell infiltration in each patient. Subsequently, the TME was characterized using the “Estimation of STromal and Immune cells in MAlignant Tumor tissues using Expression data (ESTIMATE)” algorithm. The ESTIMATE algorithm was used for calculating the tumor purity and the stromal, ESTIMATE, and immune scores. The data on the level of activation of the seven-step tumor immune cycle were retrieved from the tumor immunophenotype (TIP) (http://biocc.hrbmu.edu.cn/TIP/index.jsp) database (39). Additionally, we determined and compared the expression profile of 35 immune checkpoint genes in patients between both CDS groups to elucidate the to elucidate the ability of CDS to predict the response of patients to immune checkpoint inhibitor therapy. The data on microsatellite instability (MSI), single nucleotide variant (SNV) neoantigens, and B-cell receptor (BCR) richness, as well as T-cell receptor (TCR) richness of patients, were obtained from TCGA. The “GSEA” package was used to compare the hallmark functions and pathways enriched by patients in both CDS groups, and the reliability of the enrichment analysis was validated using the “Gene Set Variation Analysis (GSVA)” package. The gene sets with “FDR < 0.05” were considered significantly enriched.

### Predicting the patient’s response to immunotherapy and chemotherapy

To predict the responses of patients to immunotherapy, we calculated the CDS for all patients from IMvigor210 (40), GSE78220 (41), GSE79671 (42), and GSE103668 (43) cohorts. We used “Tumor Immune Dysfunction and Exclusion (TIDE)” a web-based tool for predicting the response of the patient’s to immunotherapy (44). We performed the “submap” method to determine the similarity in the expression in patients in both CDS groups and different immunotherapeutic outcomes (45). The data on drug sensitivity in cancer cell lines of human origin were downloaded from the Cancer Therapeutics Response Portal (CTRP, http://portals.broadinstitute.org/ctrp/) and Profiling Relative Inhibition Simultaneously in Mixtures (PRISM), https://depmap.org/portal/prism/) databases. We also plotted receiver operating characteristic curve (ROC) and calculated the Area Under the ROC (AUC) values for all patients using the “pRRophtic” package (46). Generally, lower AUC values indicated higher sensitivity to potential drugs (47).

### Analysis of single-cell RNA sequencing data

ScRNA-seq files of three patients with LUAD from GSE117570 were retrieved from GEO. The expression matrices were normalized using the “Seurat” package, and the top 2000 highly variable genes were identified. The batch effect was eliminated using the “harmony” package (48). The “copyKAT” and “SingleR” packages were used to annotate tumor and immune cells (49, 50). Cell clustering analysis was performed using the “T-SNE” algorithm, and the top 11 principal components were selected. Genes with “|log2FC|>1” and “adjusted *P* < 0.01” were considered marker genes.

### Tissue microarray and immunohistochemistry staining

We procured the LUAD TM (HPan-Ade060CS-01) from Shanghai Outdo Biotech Co., LTD (Shanghai, China). HLugA060PG02 contains 30 LUAD and adjacent paraneoplastic tissues. All the raw data could be obtained at the Shanghai Outdo Biotech Co. LTD’s official website. Due to the absence of two paracancer samples in TM, we only performed IHC on 30 LUAD samples and 28 paracancer samples according to the following procedure. First, TM sections were dewaxed and rehydrated using decreasing grade of ethanol solution. Next, antigen recovery was performed in an autoclave using an acidic antigen repair solution (pH 6.0), the endogenous peroxidase activity was attenuated, and the antigenic sites were blocked using 5% bovine serum albumin. TM sections were incubated with 1:200 diluted anti-DDIT4 monoclonal antibody (ProteinTech, Wuhan, China, Cat No.10638-1-AP) for 16 hours at 4°C, followed by incubation with horseradish peroxidase (Maixin, Fujian, China) conjugated secondary antibody. 3,3’-diaminobenzidine (DAB, Maixin, Fujian, China) was used for immunoreactivity, and the nuclei were counterstained with hematoxylin. Finally, Interpret the results and group the samples according to the following criteria: The appearance of faint yellow to brownish granules in the cytoplasm is considered positive, while their absence is considered negative. Staining intensity in positive samples is scored as follows: no positive staining, weakly positive: (+), yellow-brown: positive (++) and dark brown: strongly positive (+++). Expression grouping of sample: Negative and weakly positive expression is included in the low expression group, while positive and strongly positive expression is included in the high expression group.

### Cell culture and transfection

H358 and H838 (LUAD cells) and BEAS-2B (normal bronchial epithelial cells) were purchased from ATCC. All cell lines were of human origin. We cultured H358 and H838 in RPMI 1640 medium (Gibco, Shanghai, China) and BEAS-2B in DMEM (Gibco, Shanghai, China). Both mediums were supplemented with 10% fetal bovine serum (FBS, Gibco, Shanghai, China) and 1% penicillin/streptomycin. All cells were maintained in an incubator at 5% CO_2_ and 37°C. Following the guidelines specified by the manufacturer, we transfected small interfering RNA (siRNA) against *DDIT4* (*DDIT4*-siRNA) and the corresponding control siRNA (siRNA-NC) into LUAD cells at the logarithmic growth stage using Lipofectamine 3000 transfection reagent (Invitrogen, MA, USA). The siRNA sequences are shown in **Supplementary Table 3**.

### RNA extraction and real-time quantitative polymerase chain reaction

Following the manufacturer’s guidelines, we isolated total cellular RNA using an RNA extraction kit (Analytik Jena AG, Jena, Germany). Next, a Promega qRT-PCR kit (Promega, WI, USA) was used to perform reverse transcription for synthesizing cDNA using extracted RNA. RT-PCR was performed using SYBR Premix Ex Taq II (Promega, WI, USA) on a real-time PCR detection system 480II (Roche, OR, USA). The PCR reaction conditions were 1 cycle of 95°C for 2 minutes, 40 cycles of 15 seconds, 60°C for 1 minute, 1 cycle of 95°C for 15 seconds, 60°C for 15 seconds, 95°C for 15 seconds. We used β-Actin as the internal reference and the 2ΔΔCt method for quantifying relative gene expression. The primer sequences are provided in **Supplementary Table 4**.

### Cell counting kit-8 assay

We performed a CCK-8 assay (Cellcook, Guangzhou, China, Cat No. CT01A) using the manufacturer’s guidelines to determine the viability of cells transfected with *DDIT4-*siRNA and siRNA-NC. We seeded these cells in the logarithmic growth phase into 96-well plates. In order to evaluate the effect on cell proliferation capacity, 10 μl CCK-8 reagent was added in all wells and incubated for 2 hours at 37°C at 0, 24, 48, 72 h after culturing. For the sensitivity of cells to the drug, cells were treated at 37˚C with Ispinesib (0, 20, 40, 80 or 100 nM, MedChem Express, Monmouth Junction, NJ, USA, Cat No.HY-50759), Cabazitaxel (0, 10, 20, 40 or 80 nM, MedChem Express, Monmouth Junction, NJ, USA, Cat No. HY-15459) and Epothilone-b (0, 40, 80, 160 or 320 nM, MedChem Express, Monmouth Junction, NJ, USA, Cat No. HY-17029) for 24 h, respectively. 10 μl CCK-8 reagent was added in all wells and incubated for 2 hours at 37°C. Finally, we measured the absorbance of each well at 450 nm using a microplate reader.

### Clone formation assays

The clone formation rate was determined using a plate clone formation assay. 400 siRNA-NC and *DDIT4-*siRNA transfected cells/well were seeded in 12-well plates and incubated at 37°C for 14 days. Next, we washed the cells with PBS and fixed them using 4% paraformaldehyde. Finally, crystal violet was used for the purpose of staining the fixed cells, and the viable clones with a minimum of 50 cells were counted.

### Transwell assay

The invasive and migratory capacities of siRNA-NC and *DDIT4-*siRNA transfected cells were tested by Transwell (pore size 8.0 µm; Corning Inc, NY, USA) coated with Matrigel (BD Biosciences, Bedford, USA). To determine the migratory capacity of cells, we inoculated 2 × 10^4^ cells in 100 μl serum-free medium in the upper chamber. The lower chamber was supplemented with 800 μl 10% FBS-containing medium. The cells were incubated in an incubator for 24 hours, stained using crystal violet, and imaged under a light microscope. The “ImageJ” software was used for counting cells. For the invasion assay, the upper chamber was coated with 100 μl of 10% Matrigel. The rest of the procedure was the same as described above.

### TUNEL staining

We utilized TUNEL staining (Solarbio, Beijing, China, Cat No. T2196) to examine apoptosis in tumor cells. An initial density of 1 × 10^5^ cells per well was established in 12-well plates. These cells were subsequently immobilized onto coverslips with 4% paraformaldehyde for 30 minutes at room temperature, followed by two PBS washes. A treatment with 0.1% Triton X-100 was applied for 10 minutes at room temperature. After another PBS rinse, the cells were incubated in a 50 µl TUNEL reaction mixture at 37°C for 1 hour. To counterstain the cell nuclei, we employed 4,6-diamidino-2-phenylindole (DAPI, Solarbio, Beijing, China, Cat No. C0065) for 10 minutes at room temperature and washed cells twice with PBS. Finally, images of TUNEL-labeled cells were procured from three arbitrary fields using a fluorescent microscope.

### Western blotting

To obtain total protein, cells were subjected to protein extraction using 1% PMSF and RIPA buffer (Solarbio, Beijing, China, Cat No. R0020) on ice for 30 minutes. The resulting mixture was centrifuged at 12,000 rpm for 30 minutes, and the protein suspension was collected from the liquid supernatant. Protein concentration was determined using the BCA method (Epizyme, Shanghai, China, Cat No. ZJ101). Subsequently, SDS-PAGE protein loading buffer (5X) (Beyotime, China) was added to the protein suspension, followed by boiling for 10 minutes. The protein was then separated using either a 10% or 12.5% SDS-PAGE gel (Epizyme, Shanghai, China, Cat No. PG113 or PG112) and transferred onto a 0.45 μm polyvinylidene fluoride (PVDF) membrane. To block the PVDF membranes, 5% skim milk was applied for 1.5 hours. Next, the membranes were incubated with primary antibodies including *DDIT4* (ProteinTech, Wuhan, China, Cat No. 67059-1-Ig, 1:1000), *BCL2* (ProteinTech, Wuhan, China, Cat No. 68103-1-Ig, 1:1000), *Caspase-3* (Huaan, Hangzhou, China, Cat No. ET1602-39, 1:1000), and *GAPDH* (Huaan, Hangzhou, China, Cat No. ET1601-4, 1:5000), followed by incubation with corresponding secondary antibodies. Finally, the protein bands were visualized using chemiluminescence kits.

### Statistical analysis

We used software including “GraphPad Prism (version 9.00)” and “R (version 4.0.5) package” for statistically analyzing the data. We determined the correlation between two continuous variables using the “Pearson correlation”. Next, the chi-squared test was employed for comparing categorical variables, and the “Wilcoxon rank-sum” or student’s t-tests for continuous variables. All statistical tests were two-sided. If not otherwise stated, *P* < 0.05 was considered statistically significant.

## Results

### Transcriptional and genetic alterations of PCDRGs in patients with LUAD

**Figure 2:**
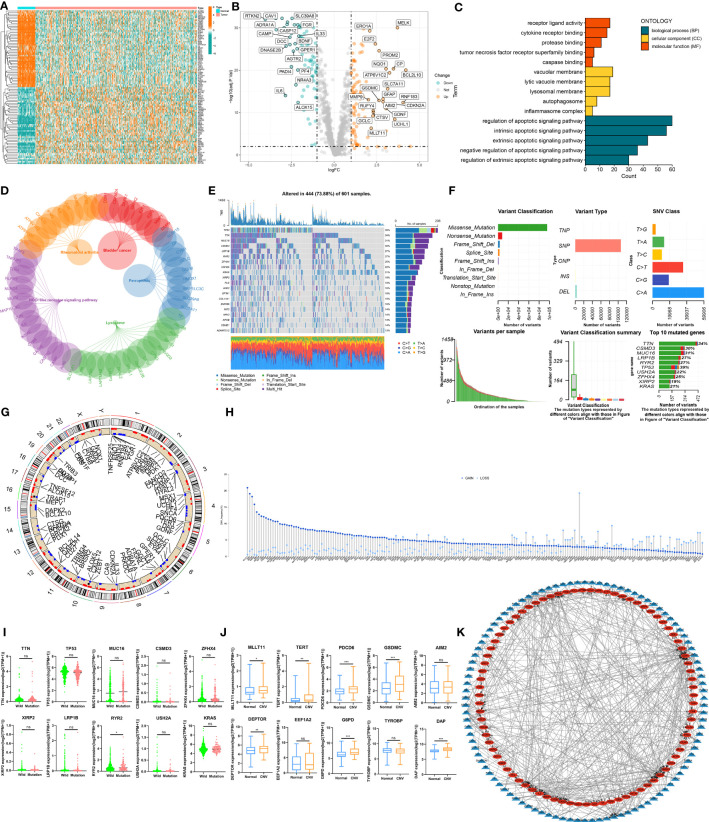
The landscape of Programmed cell death related genes (PCDRGs) in TCGA-LUAD cohort. **(A)** Heatmap of the differentially expressed PCDRGs between tumor and paracancer tissues of LUAD. **(B)** Volcano plot of the DEPCDRGs. **(C)** GO categories [molecular function (MF), biological process (BP) and cellular component (CC)] and **(D)** KEGG pathway analysis for DEPCDRGs. **(E, F)** The mutation summary and details of DEPCDRGs in the LUAD patients **(G)** The location of CNV alterations of DEPCDRGs on chromosomes. **(H)** CNV mutation situation of the DEPCDRGs. **(I)** The Scatter plot of gene expression for the top 10 DEPCDRGs with the highest CNV frequency. **(J)** Boxplot of gene expression for the top 10 DEPCDRGs with the highest mutation frequency. **(K)** Network map of the DEPCDRGs transcription factors and DEPCDRGs. Blue triangular nodes represent transcription factors, red oval nodes represent DEPCDRGs, and lines between nodes indicate regulatory relationships. ns, not significant, *P < 0.05, **P < 0.01, ***P < 0.001.

The expression profiles of DEPCDRGs between normal and LUAD tissues from the TCGA-LUAD cohort were compared, and 200 DEGs were identified (**Supplementary Table 5**). The heatmap and volcano plot shows DEGs in these samples (**Figures 2A, B**). The GO and KEGG pathway enrichment analyses showed the enrichment of these DEPCDRGs in various biological pathways like tumor necrosis factor receptor superfamily binding, the TNF, regulation of apoptotic, and IL-17 signaling pathways (**Figures 2C, D**, **Supplementary Table 6**). Next, we determined the status of DEPCDRGs mutation in patients with LUAD. Approximately 73.88% (444/601) of patients with LUAD harbored mutations in DEPCDRGs. Of the top 10 mutated DEPCDRGs, *TP53* had the highest mutation frequency (**Figures 2E, F**). The frequencies of CNV in DEPCDRGs analysis showed that most DEPCDRGs harbored significant CNVs. The chromosomal locations of CNVs in DEPCDRGs are shown in **Figure 2G**. Interestingly, the highest frequencies of CNV gain and loss were observed in *MLLT11* and *CDKN2A*, respectively (**Figure 2H**). It is worth noting that DEPCDRGs undergoing CNV often exhibit higher expression levels, but mutations and corresponding gene expression show no significant correlation (**Figures 2I, J**). 452 TF-target pairs were obtained by predicting the TFs of the genes associated with DEPCDRGs, which included 106 predicted TFs and 84 target DEPCDRGs. **Figure 2K** illustrates the regulatory relationships of these pairs.

### CDS signature development

**Figure 3:**
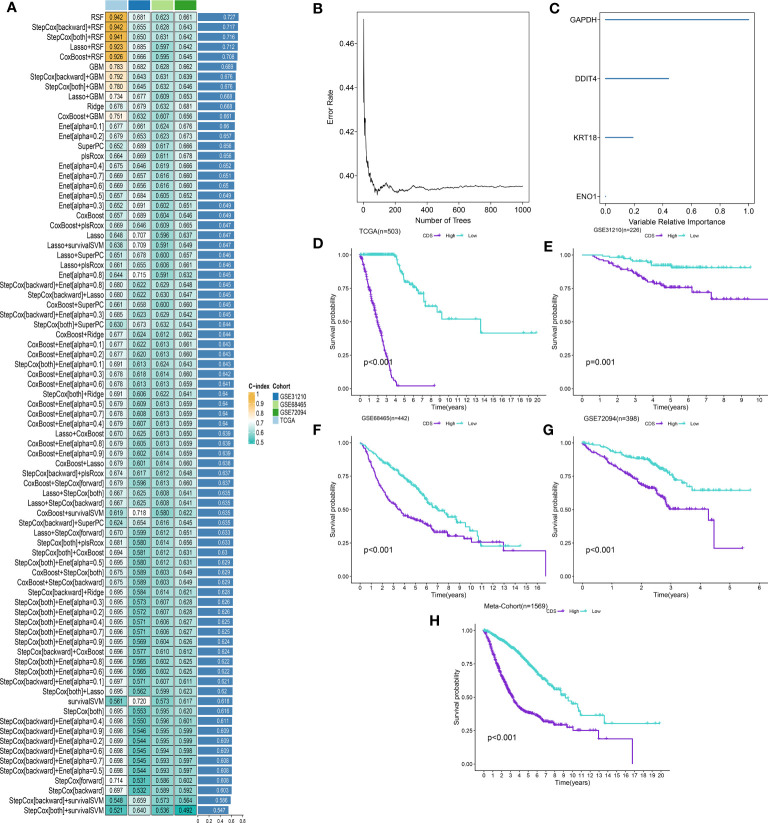
A cell death score (CDS) was established and validated *via* the machine learning-based integrative procedure. **(A)** A total of 101 kinds of machine learning algorithms were used to obtain the optimal model and calculated the C-index of each model for all cohorts. **(B, C)** The number of trees for determining the CDS with minimal error and the importance of the 4 PCDRGs based on the RSF algorithm. **(D-H)** Kaplan–Meier curves of OS according to the CDS in TCGA, GSE31210, GSE68465, GSE72094 and meta-cohort.

We performed univariate Cox regression analysis on 200 DEPCDRGs and identified 71 prognosis-related PCDRGs (**Supplementary Table 7**). These 71 PCDRGs were subjected to a machine learning-based integration procedure for developing cell death core (CDS). In addition, 101 prediction models were fitted in the training cohort using the LOOCV framework and validated on the test cohorts. Next, we calculated the C-index for all models in whole cohorts (**Figure 3A** and **Supplementary Table 8**). The mean C-index value of 0.727 was the highest in the RSF (including *GAPDH, DDIT4, KRT18*, and *ENO1*) and was considered the best model (**Figures 3B, C**, **Supplementary Table 9**). Subsequently, we calculated the CDS for all patients based on the RSF model (**Supplementary Table 10**). All patients were categorized using the median CDS value as a threshold into high and low-CDS groups. The survival duration of patients with high CDS from whole cohorts was short (**Figures 3D-H**). In addition, we evaluated the performance of CDS based on the patient’s clinical characteristics. The results demonstrated that the ability of CDS to predict patients’ survival was not influenced by their clinical characteristics (**Supplementary Figure 1**).

### CDS assessment

**Figure 4:**
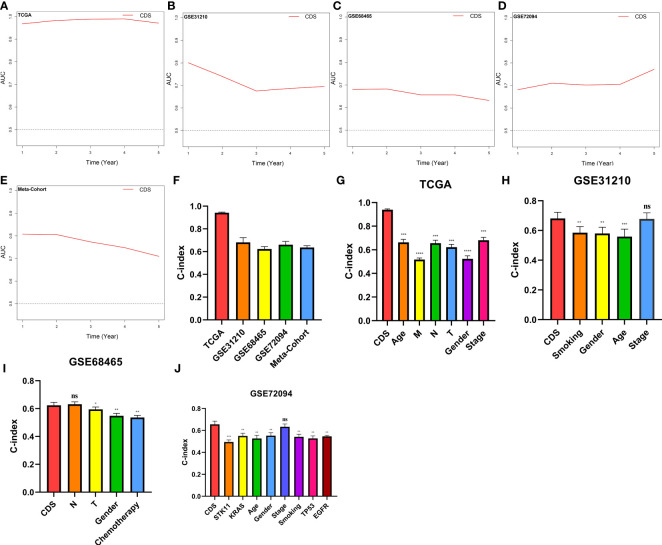
Evaluation of the CDS. **(A–E)** Time-dependent receiver operating characteristic curve of CDS for predicting the prognosis of LUAD patients from TCGA, GSE31210, GSE68465, GSE72094 and meta-cohort. **(F)** The C-index of the CDS for the TCGA, GSE31210, GSE68465, GSE72094 cohorts. **(G–J)** The C-index of the CDS and other clinical factors in the TCGA, GSE31210, GSE68465, GSE72094 cohorts. ns, not significant, *P < 0.05, **P < 0.01, ***P < 0.001, ****P < 0.0001.

**Figure 5:**
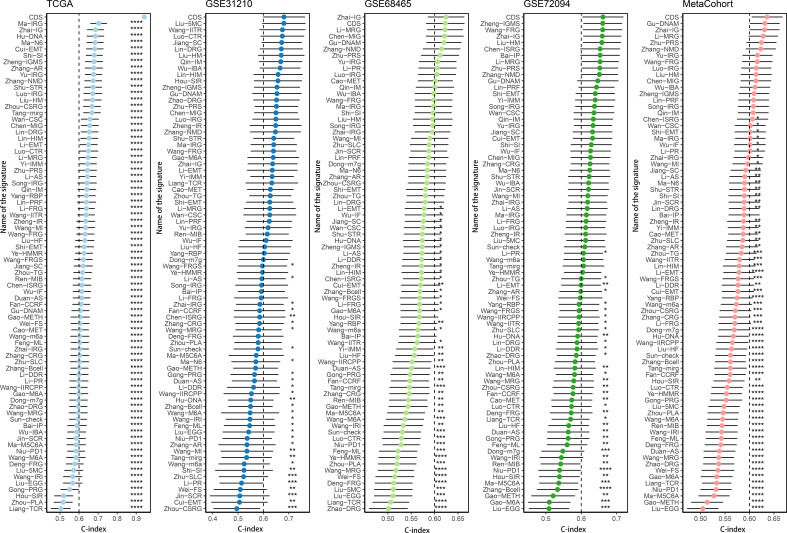
Comparison of CDS and other gene expression-based prognostic signatures in LUAD based on the TCGA, GSE31210, GSE68465, GSE72094 and meta-cohort. ns, not significant, *P < 0.05, **P < 0.01, ***P < 0.001, ****P < 0.0001.

We conducted the “time-ROC” analysis to calculate the AUC values of CDS for predicting the prognosis of patients in different cohorts [**Figures 4A-E**; TCGA [0.95–0.98], GSE31210 (0.67–0.8), GSE68465 (0.63–0.68), GSE72094 (0.68–0.78), and meta-cohort (0.71–0.81)]. The C-index values of all cohorts are shown in **Figure 4F**. Next, we compared the abilities of CDS and other clinical as well as molecular variables in predicting patients’ prognoses. The accuracy of CDS in predicting patients’ prognoses was better compared to other variables, including age, gender, smoking, *TP53, EGFR, KRAS, STK11*, M, and T (**Figures 4G-J**). The advancement in sequencing technology and bioinformatics have aided in developing models based on the combination of the expression profile of genes for predicting the patient’s diagnosis and prognosis. Subsequently, we systematically searched LUAD-related signatures published in the last 3 years. Finally, we included 77 biomarkers for comparison of predictive performance with CDS (**Supplementary Table 11**). The results revealed that the performance of our CDS in almost all cohorts was better compared to other signatures (**Figure 5**). Further, we analyzed the correlation between CDS and other clinical variables. The chi-squared test results showed a correlation between all variables except for gender and both CDS groups (**Supplementary Figure 2**). After incorporating clinical data of patients, univariate and multivariate Cox regression analyses of the four cohorts indiated that CDS could predict patients’ prognoses independently (**Tables 1**–**4**).

**Table 1:** Univariate and multivariate Cox analysis of the clinicopathological features and FA score with OS for TCGA cohort.

	Univariate Cox		Multivariate Cox	
Characteristics	HR(95%CI)	*P* value	HR(95%CI)	*P* value
Stage	1.977(1.586-2.463)	**< 0.001**	1.302(0.919-1.845)	0.137
M	1.727(1.18-2.527)	**0.005**	0.799(0.507-1.259)	0.333
N	1.942(1.575-2.394)	**< 0.001**	1.306(0.972-1.753)	0.076
T	1.816(1.386-2.38)	**< 0.001**	1.492(1.075-2.07)	**0.017**
Age	1.038(0.822-1.31)	0.754		
Sex	1.041(0.847-1.28)	0.7		
CDS	0.028(0.016-0.047)	**< 0.001**	0.035(0.019-0.062)	**< 0.001**

Significant value is given in bold.

**Table 2:** Univariate and multivariate Cox analysis of the clinicopathological features and FA score with OS for GSE68465 cohort.

	Univariate Cox		Multivariate Cox	
Characteristics	HR(95%CI)	*P* value	HR(95%CI)	*P* value
N	2.029(1.689-2.438)	**< 0.001**	2.053(1.686-2.5)	**< 0.001**
T	2.062(1.587-2.68)	**< 0.001**	1.806(1.37-2.383)	**< 0.001**
Sex	1.262(1.051-1.516)	**0.013**	1.239(1.021-1.503)	**0.03**
Chemotherapy	1.412(1.15-1.734)	**< 0.001**	1.243(1.003-1.541)	**0.047**
CDS	0.655(0.544-0.788)	**< 0.001**	0.658(0.544-0.797)	**< 0.001**

Significant value is given in bold.

**Table 3:** Univariate and multivariate Cox analysis of the clinicopathological features and FA score with OS for GSE31210 cohort.

	Univariate Cox		Multivariate Cox	
Characteristics	HR(95%CI)	*P* value	HR(95%CI)	*P* value
Smoking	1.417(0.882-2.277)	0.15	NA	NA
Sex	1.344(0.839-2.152)	0.219	NA	NA
Age	1.263(0.777-2.052)	0.346	NA	NA
Stage	2.774(1.732-4.441)	**< 0.001**	2.313(1.413-3.787)	**< 0.001**
CDS	0.434(0.254-0.743)	**0.002**	0.552(0.314-0.969)	**0.038**

Significant value is given in bold.

**Table 4:** Univariate and multivariate Cox analysis of the clinicopathological features and FA score with OS for GSE72094 cohort.

	Univariate Cox		Multivariate Cox	
Characteristics	HR(95%CI)	*P* value	HR(95%CI)	*P* value
STK11	1.028(0.72-1.469)	0.879	NA	NA
KRAS	0.767(0.588-0.999)	**0.049**	0.901(0.686-1.184)	0.454
Age	1.258(0.836-1.894)	0.27	NA	NA
Gender	0.733(0.564-0.952)	**0.02**	0.714(0.546-0.934)	**0.014**
Stage	1.969(1.477-2.625)	**< 0.001**	1.925(1.438-2.579)	**< 0.001**
Smoking	1.248(0.694-2.245)	0.459	NA	NA
CDS	0.536(0.407-0.707)	**< 0.001**	0.605(0.456-0.801)	**< 0.001**

Significant value is given in bold.

### Generation of CDS genetic subtypes

**Figure 6:**
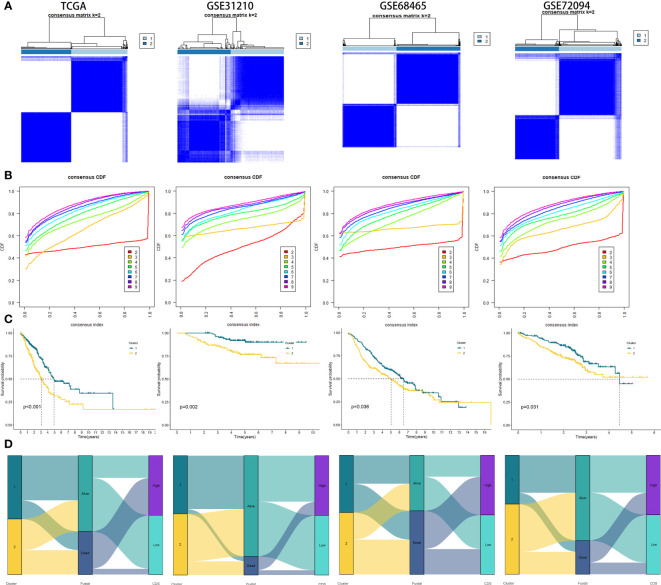
Generation of clusters by Unsupervised clustering of CDS gene expression for TCGA, GSE31210, GSE68465, GSE72094 cohorts. **(A)** Consensus clustering matrix of LUAD patients for k = 2. **(B)** Consensus clustering cumulative distribution function for k = 2 to 9. **(C)** Kaplan–Meier curves for patients in two different molecular clusters **(D)** Alluvial diagram of clusters distributions in groups with different CDS groups, clusters and survival outcomes.

We performed consistent clustering on four genes included, and the samples were grouped into distinct characteristic subtypes to identify PCD-related subtypes of LUAD. Finally, we identified two PCD-related phenotypes: clusters 1 and 2 (**Figures 6A, B**, **Supplementary Table 12**). Kaplan-Meier (KM) curves showed higher OS rate patients in cluster 1 compared to cluster 2 in all cohorts (**Figure 6C**). In addition, the alluvial diagram showed that most patients with high CDS were grouped in cluster 2 (**Figure 6D**).

### Genetic variations in CDS groups

**Figure 7:**
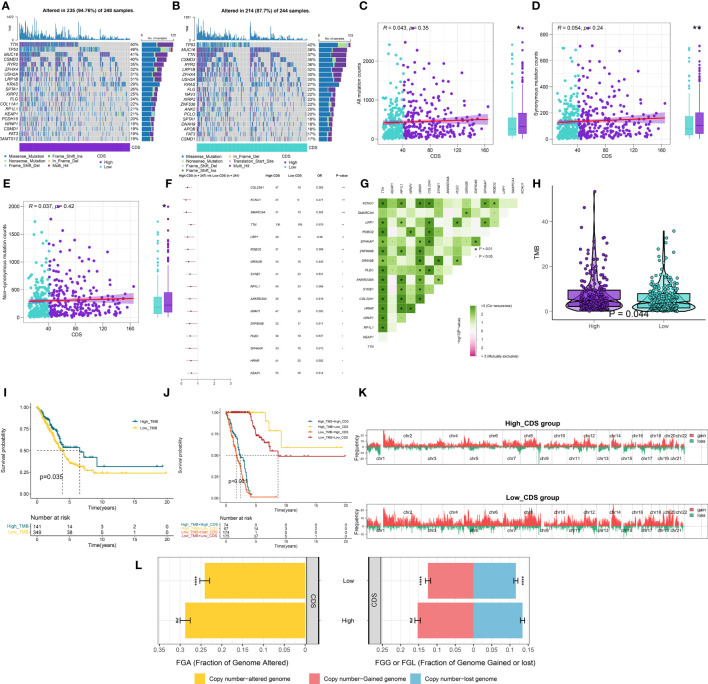
Integrated comparisons of somatic mutation and CNVs between high and low CDS groups in the TCGA cohort. **(A, B)** Waterfall plots showing the mutation information of the top 20 genes with the highest mutation frequency in the CDS groups. **(C-E)** Association between all mutation counts, synonymous mutation counts, nonsynonymous mutation counts, and CDS and their distribution in the CDS groups. **(F)** Differentially mutated genes between high and low CDS groups are displayed as a forest plot. **(G)** Interaction effect of genes mutating differentially in patients in the CDS groups. **(H)** Distribution of TMB in the CDS groups. **(I)** Kaplan–Meier curves for the OS of the high‐TMB and low‐TMB groups. **(J)** Kaplan–Meier curves for patients stratified by both TMB and CDS. **(K)** Gene fragments profiles with amplification (red) and deletion (green) among the CDS groups. **(L)** Comparison of the fraction of the genome altered, lost, and gained between the CDS groups. ns, not significant, *P < 0.05, **P < 0.01, ***P < 0.001, ****P < 0.0001.

**Figures 7A, B** shows the top 20 genes with the highest mutation frequencies in patients in both CDS groups. The results revealed differences in mutated genes in patients between both CDS groups. The frequency of sense, nonsense, or overall mutations in patients in the high-CDS group was higher compared to the low-CDS group, despite no correlation between CDS and mutation frequency (**Figures 7C-E**). In addition, a significant difference in the mutation frequency of 16 genes was observed in patients between both CDS groups (**Figure 7F**), and there was extensive co-mutation between these genes (**Figure 7G**). Patients in the high-CDS group had high TMB compared to the low-CDS group (**Figure 7H**, **Supplementary Table 13**). KM analysis showed that the OS rates of patients in the high-TMB group were significantly higher compared to the low-TMB group (**Figure 7I**). Next, the prognosis of patients was predicted based on their TMB and CDS. The survival rate of patients in the low-CDS group with high TMB was the highest, whereas the survival rate of patients in the high-CDS group with low TMB was the lowest (**Figure 7J**). CNV analysis revealed differences in chromosomal alteration patterns in patients in different CDS groups (**Figure 7K**). The high-CDS group had a greater percentage of FGA, FGL, and FGG detected. (**Figure 7L**, **Supplementary Table 14**).

### Characteristics of TME in CDS groups

**Figure 8:**
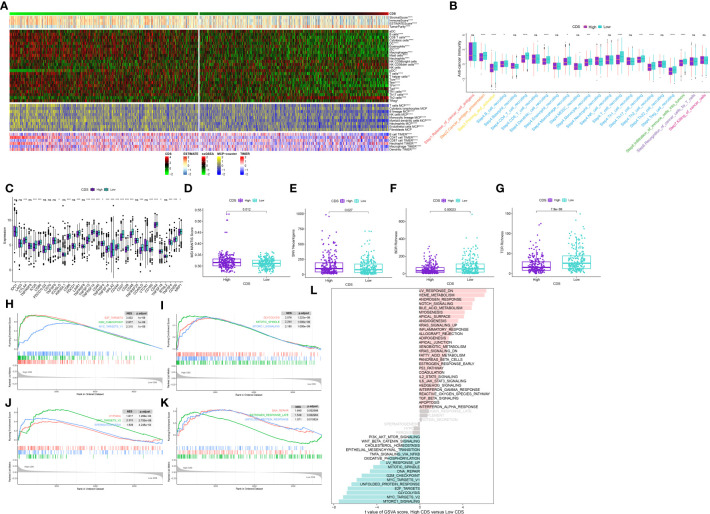
Immune-related characteristics of the CDS. **(A)** Heatmap displaying the correlation between the CDS and immune infiltrating cells in the meta-cohort. **(B)** Boxplot showing the differences of anti-cancer immunity score between CDS groups. **(C)** Comparison of immune checkpoint-related genes levels between CDS groups in the meta-cohort. **(D–G)** The distribution of MSI, neoantigens, BCR richness, TCR richness levels in different CDS groups from TCGA cohort. **(H–K)** The GSEA results for the 12 overlapping upregulated hallmark pathways in terms of the high CDS groups. **(L)** The difference in the hallmark gene sets between different CDS groups by GSVA. ns, not significant, *P < 0.05, **P < 0.01, ***P < 0.001, ****P < 0.0001.

To evaluate if CDS could be used to determine the immune status of patients, we analyzed the correlation between CDS and infiltrating immune cells (**Supplementary Table 15, 16**). The proportion of infiltrating immune cells in patients in the low-CDS group was high (**Figure 8A**). Next, our analysis of cancer progression revealed that the majority of key steps, including cancer antigen presentation, priming and activation and B cell recruiting, displayed higher activity levels in the low CDS group (**Figure 8B**, **Supplementary Table 17**). Additionally, an increase in the expression of most immune checkpoint genes was observed in patients in the low-CDS group (**Figure 8C**, **Supplementary Table 18**). Additionally, several factors associated with tumor immunogenicity were analyzed, like the status of MSI, SNV neoantigens, and BCR and TCR richness (**Supplementary Table 19**). Patients in the high-CDS group had high MSI, SNV, neoantigens, and low BCR and TCR richness (**Figures 8D-G**). Together, these results suggest that patients with low CDS had highly active immune status. GSEA analysis was performed to compare the Hallmark pathways enriched in patients in both CDS groups (**Supplementary Table 20**). We observed significant enrichment of patients with high CDS in pathways and functions related to cell cycle, hypoxia, glycolysis, and mTOR signaling, and these results were validated in GSVA analysis (**Figures 8H-L**, **Supplementary Table 21**).

### Predicting immunotherapy efficacy and identification of potential drugs

**Figure 9:**
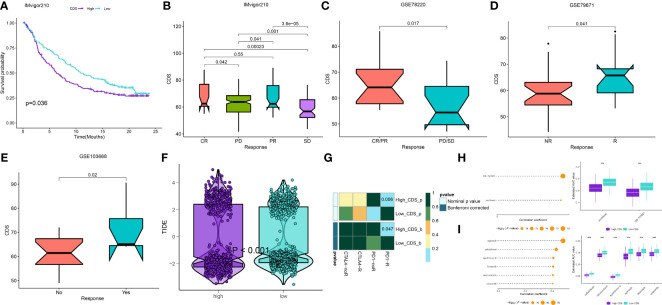
Differential putative immunotherapy and chemotherapy response for patients from high and low CDS groups. **(A)** Kaplan-Meier curve for patients in high and low CDS groups in the IMvigor cohort. **(B-E)** Box plot showing different CDS from patients with immunotherapy responses in the IMvigor, GSE103668, GSE79671 and GSE78220 cohorts. **(F)** Violin plot showing different TIDE scores from patients with different CDS. **(G)** Submap analysis of the meta-cohort and melanoma patients with detailed immunotherapeutic information. **(H)**The results of correlation analysis and differential drug response analysis of CTRP-derived drugs. **(I)** The results of correlation analysis and differential drug response analysis of PRISM-derived drugs. ns, not significant, *P < 0.05, **P < 0.01, ***P < 0.001, ****P < 0.0001.

We calculated the CDS of patients from the immunotherapy cohorts to determine if CDS could predict the response to immunotherapy. In the IMvigor210 cohort, the OS rate of patients with high CDS still was lower (**Figure 9A**), but the response of them to PD-L1 immunotherapy was better (**Figure 9B**). The response of patients from GSE78220 to immunotherapy was similar to the IMvigor210 cohort (**Figure 9C**). In addition, the response of patients with high CDS in the GSE79671 and GSE103668 cohorts to immunotherapy was better (**Figures 9D, E**). Subsequently, we analyzed patient immune evasion and immunotherapy using TIDE scores and found that patients in the high-CDS group were less likely to experience immune evasion and had better immunotherapy outcomes (**Figure 9F**, **Supplementary Table 22**). The “SubMap” algorithm results showed that patients in the high-CDS group were more likely to respond to PD-1 immunotherapy (**Figure 9G**). Next, we screened for potential drugs for treating patients with LUAD using the CTRP and PRISM-derived drug response cohorts. Finally, we obtained two compounds, paclitaxel and SB-743921 from the CTRP cohort (**Figure 9H**) and six compounds including cabazitaxel, daunorubicin, epothilone-b, ispinesib, litronesib, and volasertib from the PRISM cohort (**Figure 9I**). Interestingly, patients in the high-CDS group demonstrated sensitivity to these drugs.

### Single-cell level analysis of CDS

**Figure 10:**
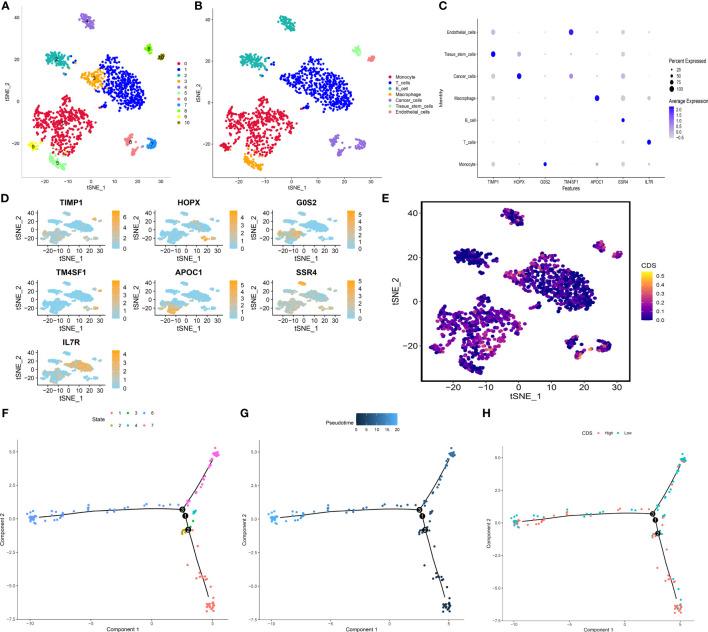
Exploration of CDS in LUAD scRNA-seq data. **(A)** t-SNE plot colored by 11 cell subpopulations. **(B)** t-SNE plot of the distribution of 7 cell types. **(C, D)** Marker gene expression of each cell type. **(E)** CDS distributions in the different single cells. **(F-H)** Pseudotime trajectory analysis in LUAD cells (Cells are colored based on states, pseudotime and CDS groups, labels 1, 2, and 3 correspond to the node identifiers and their respective quantities in the figures.

We performed principle component analysis to reduce the dimensionality of all cells using 2000 highly variable genes. Seven cell types, like monocytes, T cells, B cells, macrophages, cancer cells, tissue stem cells, and endothelial cells, were annotated (**Figures 10A, B**). Additionally, marker genes for each cell type were identified (**Figures 10C, D**), and the CDS of each cell type was calculated. Cancer cells, T cells, and monocytic regions had high CDS (**Figure 10E**). Pseudotime trajectory analysis shows the chronological order of cell differentiation. The cells with low CDS were mainly disturbed at the end of the differentiation pathway, and the cells with high CDS were primarily distributed at the early stage of the differentiation pathway (**Figures 10F-H**).

### DDIT4 affects tumor cell proliferation, invasion, migration and apoptosis

**Figure 11:**
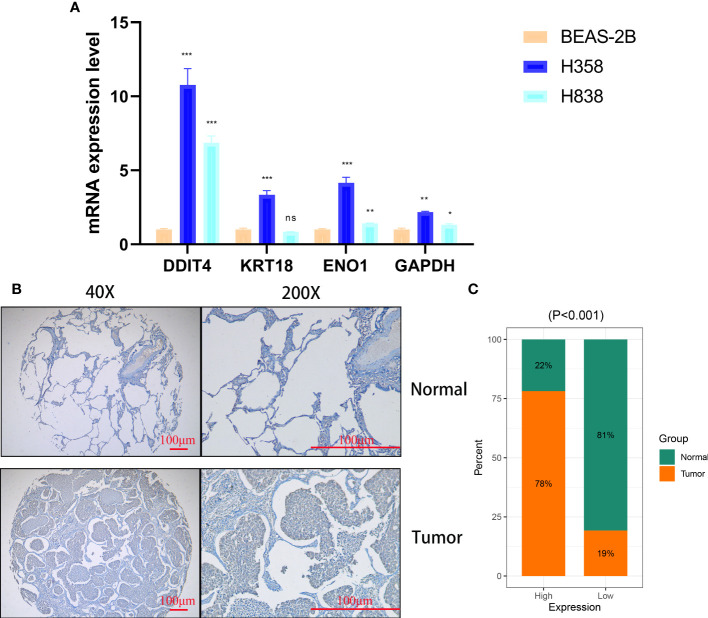
Cellular and histological and validation candidate gene expression changes. **(A)** CDS genes expression in cancer and normal cell lines. beta-actin was used as the internal reference gene and experiment was performed in triplicate and at least three times. **(B, C)** IHC analysis of DDIT4 in 30 LUAD and 28 adjacent tissues. ns, not significant, *P < 0.05, **P < 0.01, ***P < 0.001.

**Figure 12:**
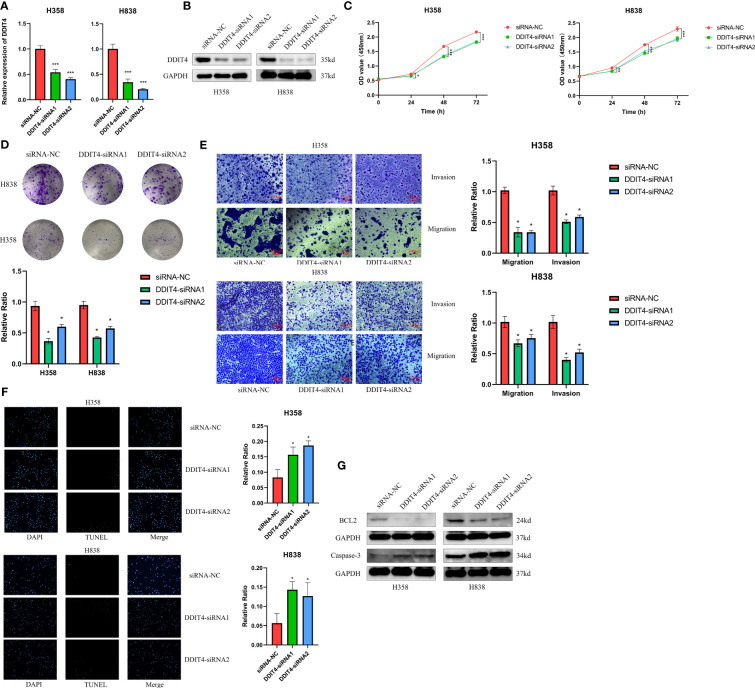
DDIT4 promoted proliferation, migration, invasion and inhibited apoptosis of LUAD cell lines. **(A, B)** Knockdown of DDIT4 was confirmed by RT-PCR and WB. beta-actin and GAPDH was used as the internal reference gene. **(C, D)** CCK8 and clone formation assays were performed to assess cell viability and proliferation of H358 and H838 cells. **(E)** Transwell assay was performed to assess cell migration and invasion of H358 and H838 cells. **(F)** TUNEL staining of H358 and H838 cells. **(G)** WB analysis of BCL2 and caspase-3 proteins in H358 and H838 cells. *P < 0.05, ***P < 0.001.

**Figure 13:**
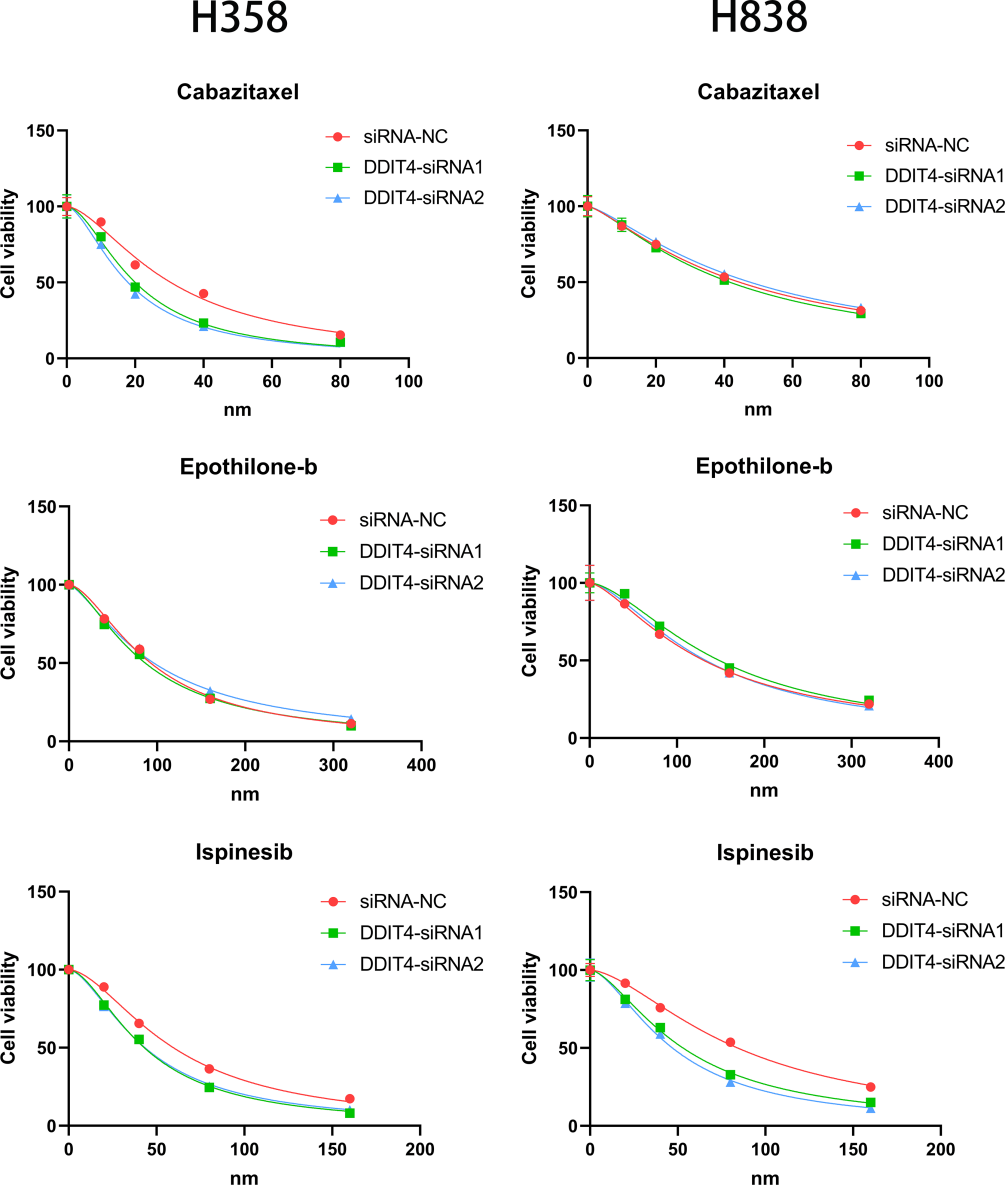
Effect of DDIT4 on chemotherapy sensitivity of H358 and H838 cells.

The expression of four genes of CDS in LUAD and normal cells was verified by RT-qPCR. Compared to normal cell lines, all genes were highly expressed in LUAD cells, with *DDIT4* showing the most significant difference (**Figure 11A**). Owing to the highest expression of DDIT4 among the four genes within LUAD cells, coupled with the absence of reports regarding its progression in LUAD, we elected to conduct subsequent experiments involving *DDIT4*. IHC confirmed high *DDIT4* expression in LUAD tissues (**Figures 11B, C**). Next, we performed several experiments to determine the roles of *DDIT4* in LUAD. RT-qPCR and WB results revealed a significant decrease in *DDIT4* expression in cells transfected with *DDIT4*-siRNAs (**Figures 12A, B**). The CCK-8 and clone formation assays showed a reduction in the viability and clone formation of cells in the *DDIT4*-siRNAs transfected cells compared to the siRNA-NC transfected cells (**Figures 12C, D**). Next, we performed transwell assay to evaluate the mobility, migratory, and invasive abilities of LUAD cells. Compared to the siRNA-NC transfected cells, a loss of invasive and migratory abilities of cells in the *DDIT4* siRNAs transfected cells was observed (**Figure 12E**). Besides, knockdown of *DDTI4* promotes apoptosis and increases sensitivity to ispinesib and cabazitaxel in LUAD cells (**Figures 12F, G** and **Figure 13**). Together, these results suggest that the *DDIT4* may play a pro-oncogenic role and a therapeutic target in LUAD.

## Discussion

Despite ongoing efforts, the treatment of LUAD remains challenging, as the disease is often aggressive and associated with poor prognosis (51, 52). Therefore, studies should focus on identifying molecular markers and therapeutic targets for LUAD. It is well established that cell death has vital anticancer effects and serves as a therapeutic target. Studies have shown that several PCDs could influence the TME and attenuate tumorigenesis, cancer progression, and cancer treatment, thus improving the prognosis and survival of patients with cancer (53, 54). Commonly used chemotherapy agents and immune checkpoint inhibitors trigger cell death, thereby attenuating cancer progression (55). However, several cancers have an innate resistance to cell death (56). Therefore, deciphering the underlying mechanisms and functions of cell death, specifically PCD types and the steps involved in regulated cell death, holds great promise for providing insights into cancer development and anti-cancer therapeutics. In clinical practice, the pathological stage of LUAD determines the patient’s prognosis (57). However, the clinical outcomes of patients with similar pathological stages of LUAD are often different, which indicates the inadequacy of current staging systems in providing reliable predictions and reflecting LUAD heterogeneity (58). As next-generation sequencing technologies continue to advance, RNA-seq has emerged as a potent approach for discovering novel biomarkers and therapeutic targets (59, 60). In recent years, numerous models based on gene assemblies of various PCD types have demonstrated commendable prognostic and therapeutic predictive value, underscoring the potent latent capabilities and clinical implications of PCD-related genes (61, 62). Nevertheless, a comprehensive analysis examining PCD-associated genes in LUAD has not yet been reported. In this study, we comprehensively analyzed PCDRGs from 12 PCD types. Using the gene expression profiles of these genes, we developed and validated 101 models through the “LOOCV” framework across multiple cohorts, resulting in the identification of the optimal RFS model. This approach not only utilizes various algorithms to fit models with consistent prognostic value for LUAD patients but also enables the models to become simpler and more interpretable. The “The “KM”, “Time-ROC” and “C-index” analyses showed higher accuracy and stability of CDS in stratifying the prognosis of patients with LUAD in multiple cohorts. Furthermore, multivariate Cox regression analysis showed that CDS could independently predict the prognosis of patients with LUAD. Next, we compared our CDS with 77 previously published genetic LUAD models, and the C-index results revealed that the performance of our CDS was better than these 77 published models. Therefore, CDS could be a novel and reliable tool for stratifying patients with LUAD.

All four PCDRGs included in CDS were associated with tumor initiation and progression. *GAPDH* is a key enzyme in step 6 of the glycolytic pathway (63). Studies have demonstrated an increase in *GAPDH* expression levels in various tumor tissues and cells (64–66). Malignant cells prefer aerobic glycolysis for producing adenosine triphosphate to oxidative phosphorylation (67). An increase in the expression of glycolytic enzymes is considered a hallmark of cancer metabolism (66). Studies have shown the involvement of *GAPDH* in several processes, like the apoptosis of cells, maintaining DNA integrity, and angiogenesis. Antisense oligonucleotides or anticancer agents targeting *GAPDH* could inhibit the proliferation of colon cancer cells and trigger the apoptosis of cervical cancer cells (68, 69). *DDIT4* is a novel HIF-responsive gene (70). Studies have shown a close association between increased *DDIT4* expression in hypoxic or stressful conditions and DNA damage, inflammation, ROS, and autophagy during cancer occurrence and development. *DDIT4* activates the TSC1/2 and NF-κB pathways, thereby endogenously inhibiting the mTORC1 pathway. High *DDIT4* expression is observed in several cancers and is linked to poor patient prognosis (71, 72). Conversely, the prognosis of patients with lung or pancreatic cancers harboring RAS mutations and *DDIT4* deletion is poor. This could be due to reprogramming the oxidation of fatty acids and the accumulation of pyruvate and lactate (73). Our *in vitro* experiments showed high *DDIT4* expression in LUAD tissues and cells, which promoted proliferation, invasion, migration of and inhibited apoptosis of LUAD cells. *KRT18* is a keratin protein and intermediate filaments necessary for tissue integrity (74). *KRT18* is one of the most abundant keratin proteins of epithelial and endothelial cells. It is expressed in many malignant tumors, including NSCLC, gastric cancer (GC), hepatocellular carcinoma (HCC) and breast cancer (BC), making it widely used as a diagnostic and prognostic marker for cancers (75–78). In addition, *KRT18* is an important regulator of tumors. *EGR1* enhances *KRT18* expression and promotes the apoptosis of NSCLC cells (76). Studies have shown that reduced *KRT18* expression enhances the susceptibility of cervical cancer cells to cytokine-induced cell death, inhibits cell migration (79), and enhances the sensitivity to paclitaxel in LC (75). *ENO1* is an enzyme involved in metabolism, the pyruvate synthesis and triggers the activation of the fibrinolytic enzyme and the degradation of the extracellular matrix (80). Several studies have demonstrated the involvement of *ENO1* in several physiological processes like metabolism, the remodeling of the extracellular matrix, controlling the growth of cells, and metastasis (81, 82). Studies have demonstrated that *ENO1* promotes the migration and metastasis of cancer cells *via* the mechanism of regulating intravascular and pericyte fibrinolytic activity (83, 84). Besides, *ENO1* could be a valuable prognostic marker. The relapse-free survival and OS of patients with NSCLC expressing high *ENO1* level is relatively shorter (85). Moreover, a study has indicated that targeting *ENO1* could be a novel and effective approach to overcoming drug resistance (86).

PCD regulates TME by triggering the crosstalk between innate and adaptive immunity to induce immunostimulatory responses (87). TME is critical for cancer development and response to treatment (88). Our results of single-cell RNA sequencing analyses and clustering showed that high CDS scoring cells are mainly concentrated in the areas of T cells, endothelial cells, and tumor cells. Monocytes have increasingly been recognized as critical influencers in cancer evolution and progression, with various subtypes displaying contradictory roles in facilitating tumor expansion and impeding the metastasis of malignant cells (89). Macrophages, notably prominent in the pulmonary cancer milieu, are significant inflammatory entities that modulate both innate and adaptive immune responses in cancer. The M1 subtype of macrophages exudes tumor-suppressing molecules like ROS and NO, eliciting cytotoxic reactions on cancer cells (90, 91). Contrarily, M2 macrophages can synthesize a range of cytokines that foster the proliferation and survival of tumor cells. Additional research indicates that an established positive feedback mechanism involving CCL5 and CCL18 between M2 macrophages and myofibroblasts contributes to the malignant progression of phyllodes tumors (92). T cells represent the predominant tumor-infiltrating immune cells in the TME (93), including various t-cell subsets. These subsets, along with select other immune cell types, perform dual roles within the lung TME, engaging in both tumor-suppressing and tumor-promoting activities (94). CD8+ T cells are pivotal in orchestrating anti-tumor immunity, effectively eliminating tumor cells through the recognition of tumor-associated antigens exhibited in major histocompatibility complex class I (95). Conversely, regulatory T cells (Tregs) are capable of suppressing anti-cancer immunity. This suppression undermines protective immunosurveillance of neoplasia and obstructs potent anti-tumor immune responses in hosts carrying tumors, thereby fostering tumor evolution and advancement (96). These cells exhibit high CDS, indicating that they may have some interaction in TME. In this study, multiple immune cells including CD4 T, CD8 T and macrophages showed high infiltration in patients with low CDS, whereas Treg cells showed low infiltration. Moreover, studies have shown that poor prognosis were closely associated with an imbalance in the ratio of immune cells in patients with cancers (97, 98). The results showed that the patients in the low-CDS group had better OS rate and higher immune scores, suggesting that patients with highly active immune state have a better prognosis. Tumor cells with lower levels of differentiation often exhibit faster growth rates, higher invasiveness, and are typically associated with poorer prognosis (99). The results of pseudotime analysis indicated that tumor cells with high CDS levels were positioned at the front end of the differentiation pathway, while tumor cells with low CDS levels were located at the terminal end of the differentiation pathway. Therefore, we found that the levels of CDS may be associated with the degree of differentiation and invasiveness in tumor cells. CNV is a prevalent type of variation in tumors and serves as a pivotal factor propelling the initiation and progression of cancer. Studies suggest that elevated levels of CNV can stimulate tumor cell proliferation and immune evasion, often resulting in a poorer prognosis for patients (100, 101). The levels of FGA, FGG, and FGL were significantly high in patients in the high-CDS group in our study, which corroborates previous research reports. Numerous studies suggest that patients with higher levels of TMB, MSI, and SNV neoantigens are more likely to respond to immune therapy, while those with higher TIDE scores tend to exhibit the opposite trend (102–105). Therefore, we compared TMB, MSI, SNV neoantigen, and TIDE scores in patients in both CDS groups to predict patients’ response to immunotherapy. As expected, patients in the high-CDS group had higher TMB, MSI, SNV neoantigens, and lower TIDE scores. Furthermore, patients in the high-CDS group responded better to immunotherapy and could gain more benefit from immunotherapy in multiple cohorts. These results validate the efficacy of our CDS in predicting patients’ responses to immunotherapy. Several studies are focusing on the combined use of chemo and immunotherapies for treating patients with cancer. Immunotherapy can reduce damage caused to the immune system by chemotherapy, and the combined use of chemo and immunotherapies could exert synergistic antitumor effects (106, 107). Finally, we performed an interaction analysis between CDS and drug response to screen for drugs that can be used in combination with immunotherapy in patients in the high-CDS group and aid in guiding personalized therapy. As a result, 8 potential anti-tumor drugs that are more sensitive to patients with high CDS were identified. Ispinesib is a highly selective small molecule inhibitor of KSP that inhibits the formation of bipolar mitotic spindles, leading to cell cycle arrest without centrosome separation (108). It exhibits broad-spectrum antitumor activity in various *in vitro* tumor cell lines and xenograft models. Cabazitaxel is a chemotherapy drug approved for the treatment of prostate cancer, primarily exerting its antiproliferative effect by inhibiting spindle formation and function (109). Cabazitaxel exhibits broad-spectrum antitumor activity against various tumors, including Furthermore, colorectal cancer, pancreatic cancer, and lung cancer (110). Cabazitaxel promotes autophagic cell death in LUAD by targeting the PI3K/Akt/mTOR pathway (111). Moreover, self-assembled micelles loaded with cabazitaxel exhibit good hydrophilicity and enhanced anticancer effects, making them potential candidates for lung cancer treatment (112). Similar to cabazitaxel, epothilone-b belongs to the class of microtubule stabilizers. Epothilone-b exerts its anticancer effect mainly by activating the extrinsic apoptosis pathway involving caspase-3 and caspase-8 (113). Furthermore, epothilone-b has been confirmed as one of the clinical drugs capable of inducing genuine immunogenic cell death (114). For lung cancer, epothilone-b enhances the radiosensitivity of LUAD cells by reducing DNA repair capacity (115). However, possibly due to the limitations of cell line types, our experimental results found that knockdown of DDIT4 can only stably affect the sensitivity of LUAD cells to ispinesib. Moreover, the results of GSEA and GSVA analyses indicate a significant enrichment in cell proliferation and metabolism in patients with high CDS, such as “OXIDATIVE_PHOSPHORYLATION”, “DNA_REPAIR”, “G2M_CHECKPOINT”, and “GLYCOLYSIS” (116–119). This could potentially elucidate the heightened sensitivity of patients in the high-CDS group to these chemotherapeutic drugs.

However, our study has several limitations. Firstly, due to the fact that research on PCD is a rapidly evolving and emerging field, it is possible that an increasing number of PCDRGs will be identified beyond the 1215 genes included in this study. Second, the patients included in our study were from retrospective studies conducted at single centers. Therefore, prospective studies at multiple centers should be conducted to validate the reliability and validity of CDS. Finally, we have only explored the effect of *DDIT4* on LUAD cells using siRNA. Therefore, more genetic modification and intervention strategies are required to determine the involvement and mechanism of *DDIT4* in LUAD.

## Conclusions

In conclusion, we have developed and validated an accurate and robust CDS based on four PCDRGs using extensive machine learning algorithms. Our CDS could effectively predict the survival and response of patients with LUAD to immunotherapy. CDS is a powerful tool for predicting the patient’s prognosis and designing personalized therapy. These results provide new directions and shed light on the molecular mechanisms of LUAD.

## Data availability statement

The datasets presented in this study can be found in online repositories. The names of the repository/repositories and accession number(s) can be found in the article/**Supplementary Material**.

## Ethics statement

The studies involving humans were approved by Ethics Committee and Institutional Review Board of the Outdo Biotech. Co., Ltd. (SHYJS-CP-1904007). The studies were conducted in accordance with the local legislation and institutional requirements. The participants provided their written informed consent to participate in this study.

## Author contributions

YH conceived and designed the study. YZ and YW performed the collection and assembly of data. JC and YX analyzed the data. YZ and YW performed experiments and wrote the manuscript. All authors read and approved the final manuscript.
