# Supplementary material for: A programmed cell death-related model based on machine learning for predicting prognosis and immunotherapy responses in patients with lung adenocarcinoma
Source: Front Immunol. 2023 Aug 21;14:1183230. doi: 10.3389/fimmu.2023.1183230 (PMC10475728; doi:10.3389/fimmu.2023.1183230)
Supplement: Supplementary file 1 [file DataSheet_1.pdf]

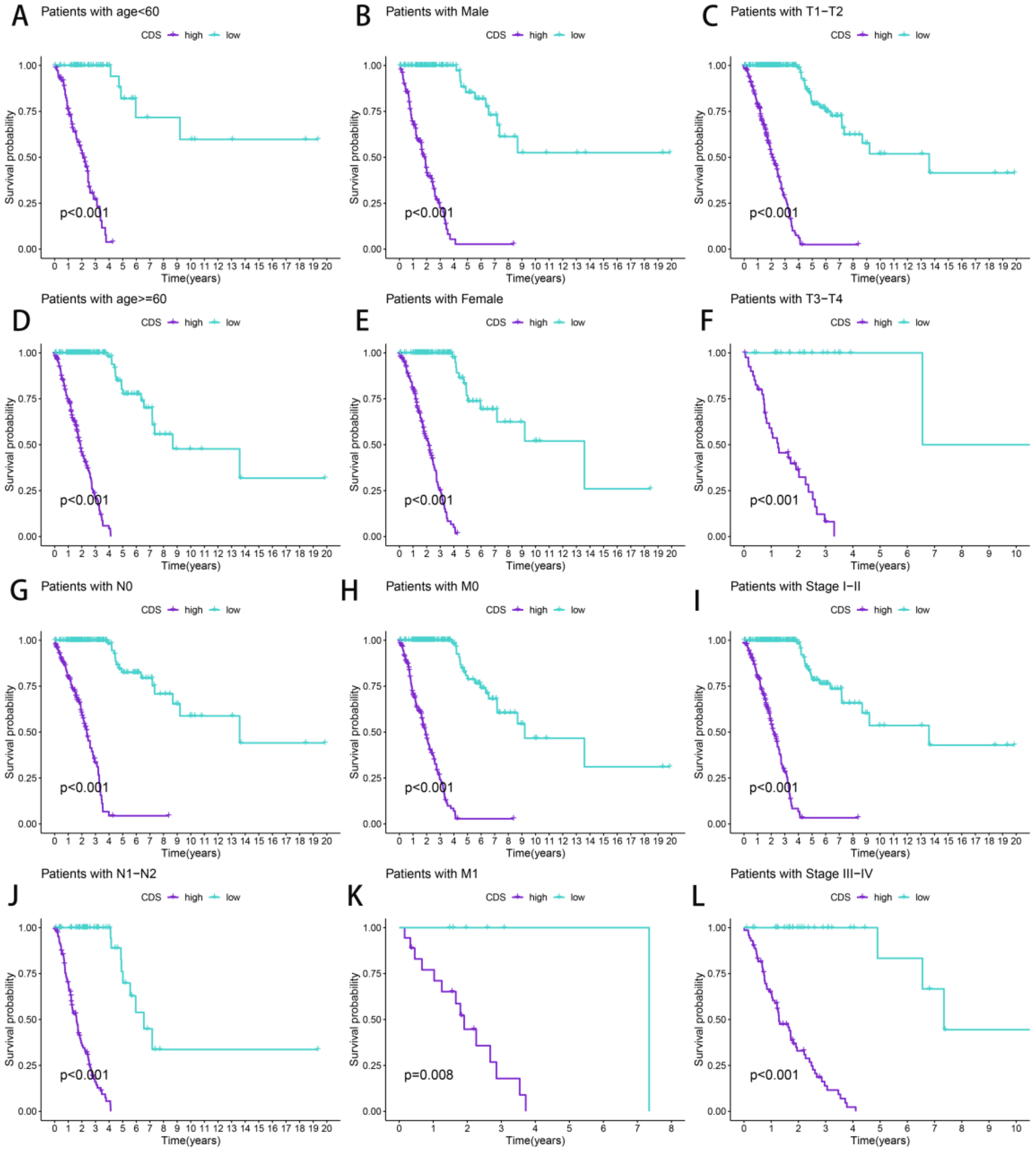

Supplementary Figure 1. The survival curves of the FA score stratified by age, gender, T, N, M and stage. (A) ≥60 years, **(B)** female, **(C)** T1-2, **(D)** <60 years, **(E)** male, **(F)** T3-4, **(G)** N0, **(H)** M0, **(I)** stage1-2, **(J)** N1-2, **(K)** M1, **(L)** stage3-4.

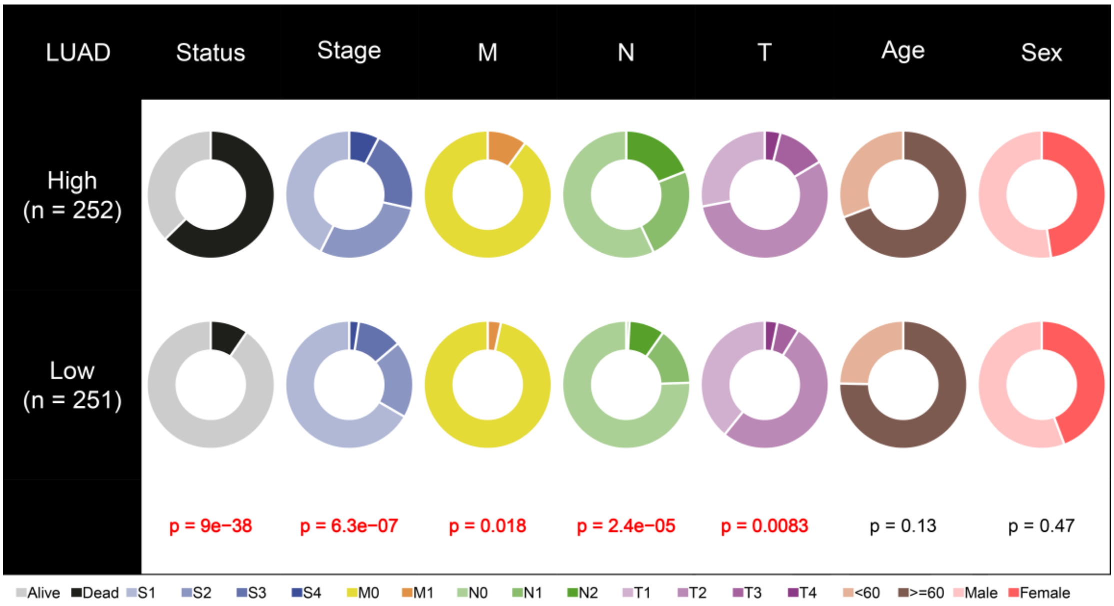

Supplementary Figure 2. The circular pie chart for the proportion difference of clinical indices.
